# A comparative study of international and Chinese public health emergency management from the perspective of knowledge domains mapping

**DOI:** 10.1186/s12199-020-00896-z

**Published:** 2020-10-02

**Authors:** Juan Li, Yuhang Zhu, Jianing Feng, Weijing Meng, Kseniia Begma, Gaopei Zhu, Xiaoxuan Wang, Di Wu, Fuyan Shi, Suzhen Wang

**Affiliations:** 1 School of Public Health, Weifang Medical University, Weifang, 261053 People’s Republic of China; 2 Center for Psychosocial Medicine, University Medical Center Hamburg-Eppendorf, 20246 Hamburg, Germany; 3 School of Life Science and Technology, Weifang Medical University, Weifang, 261053 People’s Republic of China; 4 School of Foreign Languages, Weifang Medical University, Weifang, 261053 People’s Republic of China; 5 Department of Teaching Quality Monitoring and Evaluation, Weifang Medical University, Weifang, 261053 People’s Republic of China

**Keywords:** Public health, Emergency management, Knowledge domains

## Abstract

**Background:**

At the end of 2019, the outbreak of coronavirus disease 2019 (COVID-19) severely damaged and endangered people’s lives. The public health emergency management system in China has played an essential role in handling the response to the outbreak, which has been appreciated by the World Health Organization and some countries. Hence, it is necessary to conduct an overall analysis of the development of the health emergency management system in China. This can provide a reference for scholars to aid in understanding the current situation and to reveal new research topics.

**Methods:**

We collected 2247 international articles from the Web of Science database and 959 Chinese articles from the China National Knowledge Infrastructure database. Bibliometric and mapping knowledge domain analysis methods were used in this study for temporal distribution analysis, cooperation network analysis, and co-word network analysis.

**Results:**

The first international article in this field was published in 1991, while the first Chinese article was published in 2005. The research institutions producing these studies mainly existed in universities and health organizations. Developed countries and European countries published the most articles overall, while eastern China published the most articles within China. There were 52 burst words for international articles published from 1999–2018 and 18 burst words for Chinese articles published from 2003–2018. International top-ranked articles according to the number of citations appeared in 2005, 2007, 2009, 2014, 2015, and 2016, while the corresponding Chinese articles appeared in 2003, 2004, 2009, and 2011.

**Conclusions:**

There are differences in the regional and economic distribution of international and Chinese cooperation networks. International research is often related to timely issues mainly by focusing on emergency preparedness and monitoring of public health events, while China has focused on public health emergencies and their disposition. International research began on terrorism and bioterrorism, followed by disaster planning and emergency preparedness, epidemics, and infectious diseases. China considered severe acute respiratory syndrome as the starting research background and the legal system construction as the research starting point, which was followed by the mechanism, structure, system, and training abroad for public health emergency management.

## Background

Public health emergencies have increased in recent years and have shown a trend of causing considerable damage [1]. According to the emergency events database (EM-DAT), the most widely used and influential disaster database in the world, the average number of deaths per major public health event was more than 10,000 [2]. Population growth, urban development, migration, and other issues brought about by globalization have sped up the incidence of public health events, such as epidemics [3, 4]. Public health events also propelled the process of emergency management, giving top priority to changes in emergency operations. The outbreak of coronavirus disease 2019 (COVID-19) in China spread rapidly throughout the world in a short time, which illustrated the need to build a resilient health emergency system that can withstand epidemics [5].

Public health emergency management (PHEM) is a relatively new field that draws on specific sets of knowledge, techniques, and organizing principles found in emergency management [6]. Specifically, it includes public health emergency planning, organization, leadership, coordination, control, evaluation, prevention, preparation, and response [7]. For COVID-19, China’s PHEM system quickly took the following measures: emergency mobilization measures within the government, lockdown of cities and communities, nationwide medical mobilization, provision of financial support, preferential policies for the medical community and pharmaceutical industry, and the categorical comprehensive publicity to spread prevention and treatment knowledge [8]. These measures effectively reduced the spread of the disease. Thus, current recommendations are mostly derived from the reported Chinese experience [9]. Given the weaknesses and deficiencies exposed by the COVID-19 outbreak, people have recognized the need to improve the national PHEM system [10, 11].

A growing body of research has studied PHEM from different perspectives, mainly those of institutions, funds, technologies, and laws. The public health emergency was a severe challenge to health institutions such as hospitals, the Centers for Disease Control and Prevention (CDC), and governments [7, 12]. The solutions to these challenges were characterized by sustainability, redundancy, and flexibility [13]. Monetary and technology resources can merge the roles and responsibilities of public health preparedness and emergency management [14]. Severe deficiencies in legal preparedness can undermine effective responses to public health emergencies [15, 16]. These were the essential factors in dealing with public health emergencies. Additionally, many countries took corresponding measures to strengthen the emergency management of public health. For example, the USA established PHEM operations centers either independently in health departments or as a part of the overall command system in the government [17]. China established the PHEM system from the national level to the local level to be responsible for emergency preparedness and response in 2004 [17]. In March 2018, the Ministry of Emergency Management of the People’s Republic of China was established, which was an integral part of the State Council. Thus, we can see that PHEM is still a timely topic for scholars and governments [18, 19].

However, there are still some problems that need to be solved. To the best of our knowledge, there is little evidence about the differences that occurred between international and Chinese PHEM. Moreover, what are the hotspots and trends of PHEM? What are the main research forces of PHEM? It is necessary to sort out the characteristics of the development of PHEM and explore the hotspots of PHEM research. Additionally, we compared international and Chinese research on PHEM. Based on this situation, we reviewed the articles on PHEM that were published over the past nearly 30 years in international and Chinese journals. Then, we used the knowledge map method to reveal the research strengths, frontiers, and development trends in this field. Study conclusions are helpful to draw people’s attention to public health emergencies, provide a reference for scholars to understand the current situation and trends of PHEM, and for government departments to formulate guidance strategies.

## Methods

### Data sources

Data were divided into two categories: international and Chinese data. According to the relevant authoritative research [20], the database of bibliometric methods should contain complete documents. A considerable amount of literature has shown that the Web of Science (WoS) is the largest comprehensive academic information resource, covering peer-reviewed journals with high impact factors [21–24]. Accordingly, the international data used for our study were collected from the WoS Core Collection, including Science Citation Index Expanded (SCI-E), Science Citation Index Expanded (SSCI), and Arts & Humanities Citation Index (A&HCI) databases. Chinese data were downloaded from the China National Knowledge Infrastructure (CNKI), which had the largest Chinese journal full-text database, including the vast majority of Chinese journals relating to public health management. More importantly, it has become one of the critical basic data sources for bibliometric research in China [25].

### Data retrieval

Data obtained by inappropriate literature information retrieval strategies could not accurately reflect the content of the research [26]. Emergency management is a common term in China that focuses on the occurrence, development, and evolution of emergencies and finding effective ways of responding to them [27]. However, it was not certified internationally. After consulting the experts from the Chinese CDC who once worked for the World Health Organization (WHO), we learned that the management was refined by preparedness, operation, response, and recovery for an international public health emergency. Additionally, this has been mentioned in articles [5, 28]. Based on the above points, the international data retrieval strategies were set as: ((TS = public health) AND (TS = preparedness OR TS = operation OR TS = response OR TS = recovery) AND TS = (emergency)) AND LANGUAGE: (English) AND DOCUMENT TYPES: (Article) Indexes = SCI-EXPANDED, SSCI, A&HCI, Timespan = 1988–2018. When retrieving Chinese data, we choose “public health” and “emergency management” as the theme words, Timespan = 1988–2018. We ran the search query of WoS and CNKI on February 19, 2019. A total of 2759 articles from 1991 to 2018 were retained from WoS, while 999 articles from 2003 to 2018 were retained from CNKI.

**Fig. 1:**
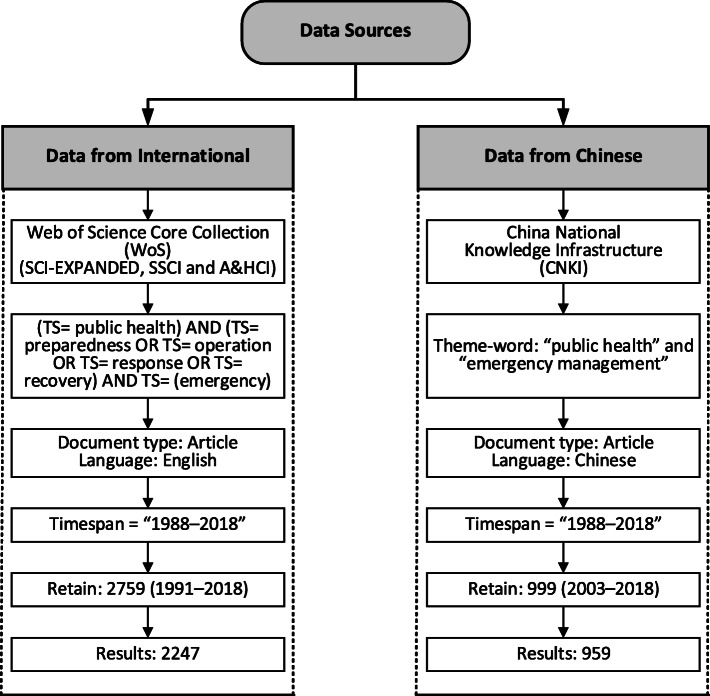
Retrieval procedure of study data

After discussing the results with the team members, we further selected the articles based on inclusion and exclusion criteria to ensure that all of the data were closely aligned to the study targets. The inclusion criteria were as follows: (1) occurrence, development, and evolution of PHEM; (2) prevention, preparedness, response, operation, and recovery of the PHEM system; (3) planning, organization, leadership, coordination, and control of public health emergencies; and (4) practice and method of PHEM. The exclusion criteria were as follows: (1) health care, medical record management, or disease treatment; (2) guidelines on the action, proceedings paper or book chapter, interviews, summaries of conferences, and patent abstracts. Finally, 2247 international articles and 959 Chinese articles were accepted for the analysis after data filtering and removing duplications. The international articles were downloaded in “plain text” format with a full record and cited references for classification and statistical analysis. The Chinese articles were downloaded in the “Refworks” format. Those downloaded data contained the list of authors, the title of the publication, the abstract, keywords, and so on. Accordingly, we obtained the data for this study. Figure 1 shows the specific search steps.

### Data analysis tools

We used CiteSpace 5.5. R2 and Microsoft Excel 2016 for the data analysis. CiteSpace was a free Java-based application found by Chaomei Chen to analyze the potential knowledge contained in the scientific literature. It has been widely adopted for scientometric analysis in various scientific fields [29], and has achieved excellent results [30, 31]. The parameters of CiteSpace for this study were set as follows: time slicing (1991–2018, 2003–2018, respectively), years per slice (1), term source (all selection), node type (author, institution, keyword, for Chinese and international data; country for international data), selection criteria (top 50), and visualization (cluster view-static, show merged network). The corresponding other settings were selected according to different study questions. Microsoft Excel 2016 was used for temporal distribution and polynomial prediction of the number of articles. It should be noted that the results of the CNKI database made by CiteSpace were presented in Chinese. To make it easier to read, we translated the Chinese results into English.

### Data analysis strategies

This study involved using bibliometric and mapping knowledge domain analysis methods. Bibliometric methods provided an approach to identify the development trends or future research orientations by combining different tools and methods to analyze the articles [32, 33]. It allowed researchers to generate information from historical data and indicators, such as keywords, authors, institutes, and countries [34]. We were mainly engaged in cooperation network analysis (including the network analysis of the authors, institutions, and countries) and co-word network analysis (including keyword co-occurrence network and burst detection analysis) in the mapping knowledge domain analysis. First, we performed a statistical analysis of the temporal distribution of relevant articles. Then, we made a polynomial prediction of the number of articles, fitted the trend line of international and Chinese study, and predicted it for the next 3 years. Cooperation network analysis was used to analyze the contribution to different authors, institutions, and countries in one field. It was obvious that the more an author, a country, or an institution publishes its research findings, the more contributions it will make [35]. Betweenness centrality is an index to measure the importance of nodes in the network. The purple circle represents documents with betweenness centrality not less than 0.1, which means that the authors, institutions, or countries occupied an essential position in this field [20, 36]. Co-word analysis was a content analysis technique that was effective for mapping co-occurrence relationships and the strength of the relationship between a pair of items existing in the same text, revealing the inner construction of a research field [24]. Analysis of the keyword co-occurrence network was meaningful and valuable for exploring timely topics in a specific knowledge domain [37, 38]. In addition, keyword burst detection analysis can clearly grasp articles that receive particular attention from related scientific communities in a certain period. Therefore, the frontiers founded by burst detection analysis can provide researchers with up-to-date information [39, 40]. The analysis of the highly cited articles in the field can reflect the development of the discipline in a period, examine timely topics, and supplement the above results to provide a reference for topic selection by scientific researchers [41].

## Results

### Temporal distribution analysis

**Fig. 2:**
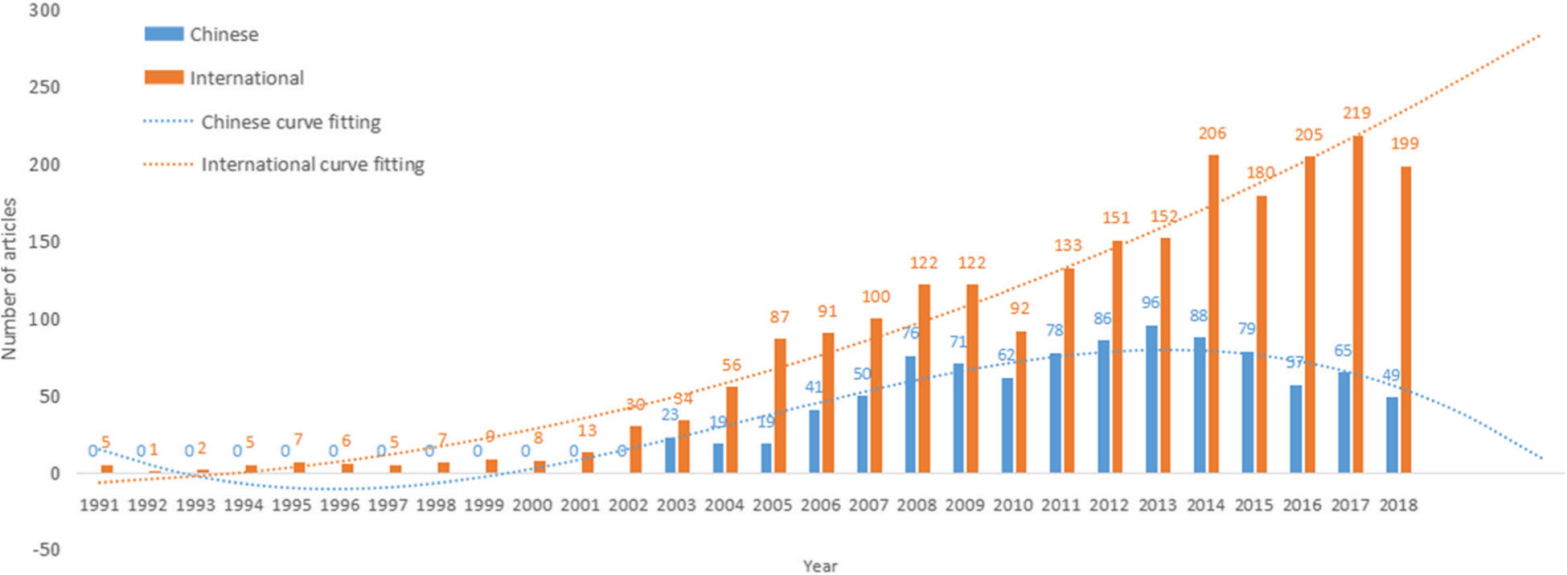
Temporal distribution and trend of international and Chinese PHEM study

Figure 2 shows the first international article on PHEM published in 1991. The number of articles fluctuated slowly over the next few years. After 2001, the number began to increase rapidly. It is worth noting that the number of articles in 2014 was high, reaching 206. It declined over the next 2 years until 2017, when the number of articles reached its peak at 219. The study on PHEM in China started in 2003 and declined slowly until 2005. During 2006–2013, there was a rapid development; many articles appeared in this period. The minimum number, in 2006, was 41, and the maximum was in 2013, reaching an astonishing 96. The average number peaked at 70. The number of articles declined after 2013, reaching 49 in 2018. In contrast, the study of PHEM in China started later than in some other countries, yet a large number of Chinese scholars participated in this study at the beginning.

After the descriptive analysis of the data, we conducted a polynomial prediction analysis of the number of articles and predicted it for the next 3 years. The trend of chronological distribution of articles related to PHEM in international data can be expressed as follows: *y* = 0.2790*x*^2^ + 0.7688*x*−7.7708 (*R*^2^ = 0.9577), while Chinese data was *y* = −0.0345*x*^3^ + 1.5079*x*^2^−14.142*x* + 27.273 (*R*^2^ = 0.9251). *Y* indicates the number of articles, and *x* indicates the years. *R*^2^ > 0.9, indicating a good degree of fit. The chronological distribution of international articles showed a trend of increasing year by year. In 2019–2021, the annual number of articles will exceed that of previous years. At the same time, the trend line of Chinese articles, such as a wave line, and the number of articles will continue to decrease over the next 3 years. In addition, as time goes on, the gap in the number of articles in Chinese and international journals will be gradually increasing.

### Cooperation network analysis

**Fig. 3:**
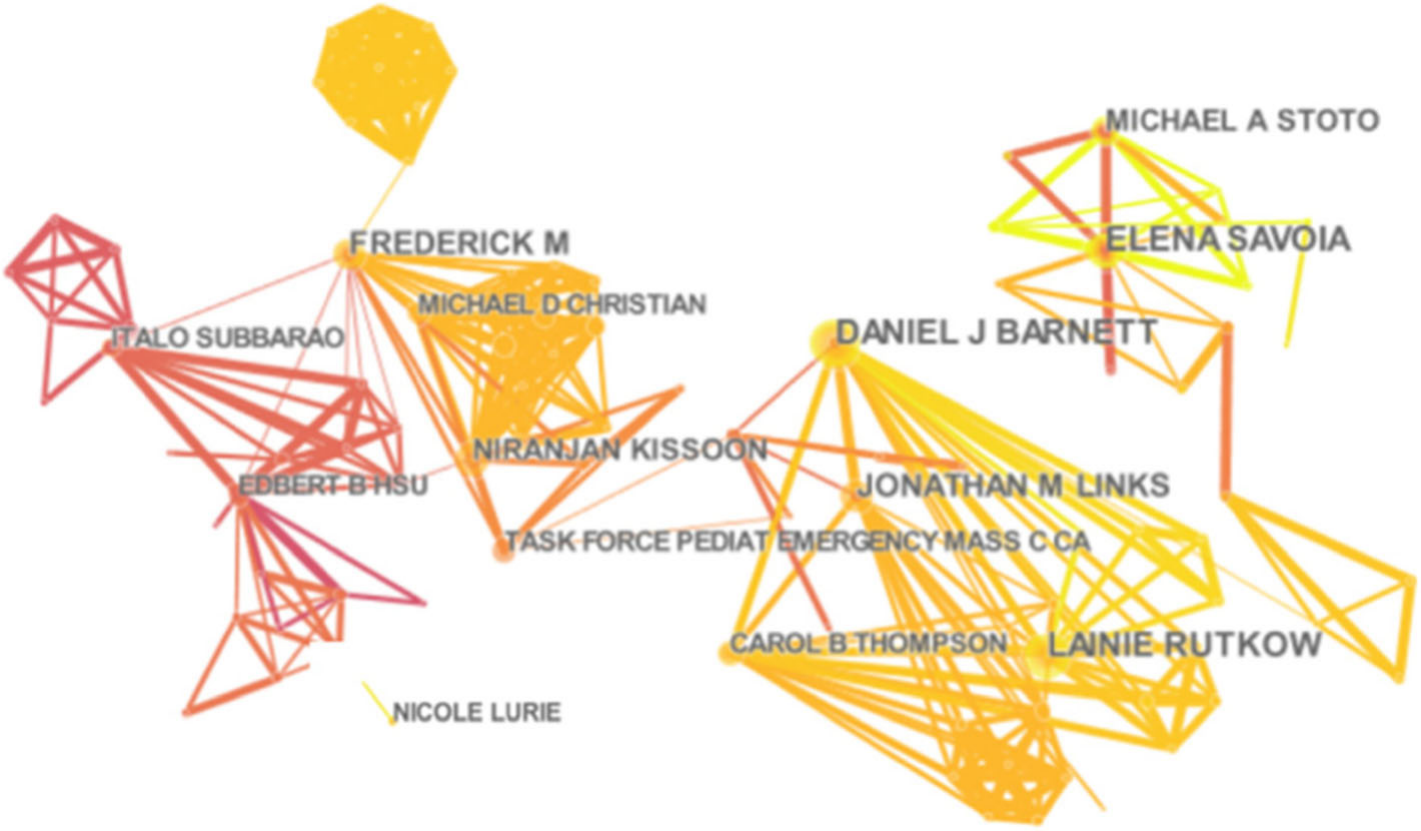
Co-author network of international database. Different colors represent different time slices. The size of node represents the number of articles by the author. The link strength between two nodes means the collaboration intensity between authors

**Fig. 4:**
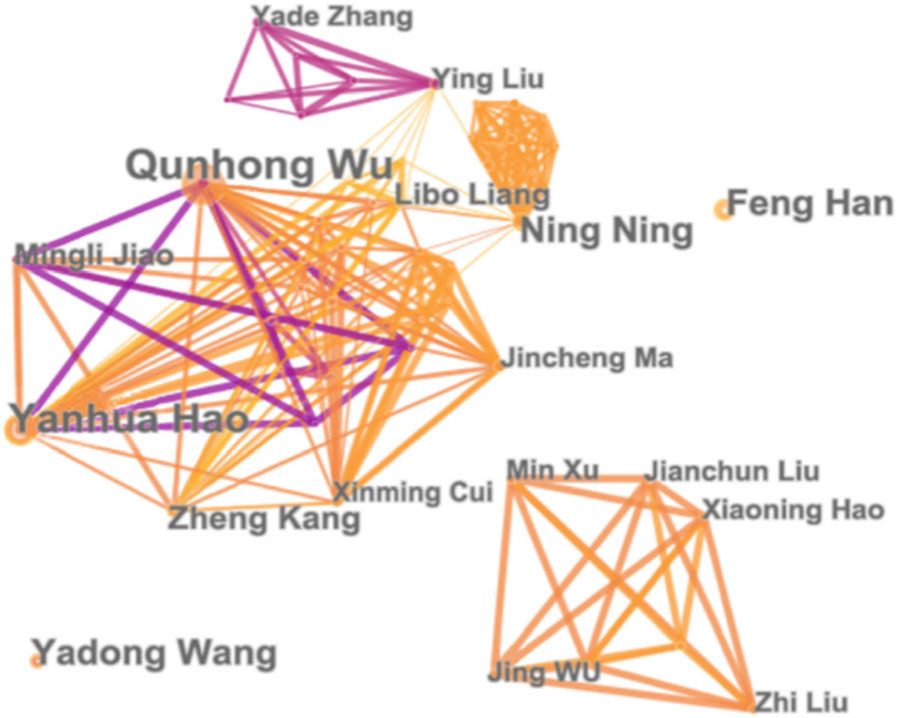
Co-author network of Chinese database. Different colors represent different time slices. The size of node represents the number of articles by the author. The link strength between two nodes means the collaboration intensity between authors

Figure 3, which shows the international co-author network, shows that there have been many authors writing studies on PHEM, and some had collaborators. The top-ranked item by citation count was Daniel J. Barnett (DANIEL J BARNETT in the figure), with a citation count of 20 (all counts are not shown in the figure). He was followed by Elena Savoia (ELENA SAVOIA) and Lainie Rutkow (LAINIE RUTKOW); both of their citation counts were 17. These were authors who paid great attention to this field and had published the most articles. Most of these authors were from American universities, such as Johns Hopkins University and Harvard University. The top-ranked items by centrality were Frederick M. Brokle (FREDERICK M), Task Force for Pediatric Emergency Mass Critical Care (TASK FORCE PEDIAT EMERGENCY MASS C CA), and James G. Hodge (JAMES G), with a centrality of 0.06 (all centralities are not shown in the figure). However, all three items’ centrality was less than 0.1. In Fig. 4, the top-ranked item for Chinese scholars by citation count was Qunhong Wu with a citation count of 22. She was followed by Yanhua Hao (17), Feng Han (11), Ning (10), Yadong Wang (10), Zheng Kang (8), Ying Liu (6), Jincheng Ma (6), Mingli Jiao (5), and Libo Liang (5). Most of them were teachers at Harbin Medical University. The top-ranked items by centrality were Yanhua Hao and Ning, with a centrality of 0.02. The third one was Qunhong Wu, with a centrality of 0.01. Their nodes’ centrality is also less than 0.1. In terms of the quantity and quality of articles, Qunhong Wu and Yanhua Hao were the leaders of PHEM in China. From the co-author network, we can see that many authors internationally and those in China are collaborating on PHEM (Additional file 1: Table S1, Table S2, and Figure S1).

### Co-institution

**Fig. 5:**
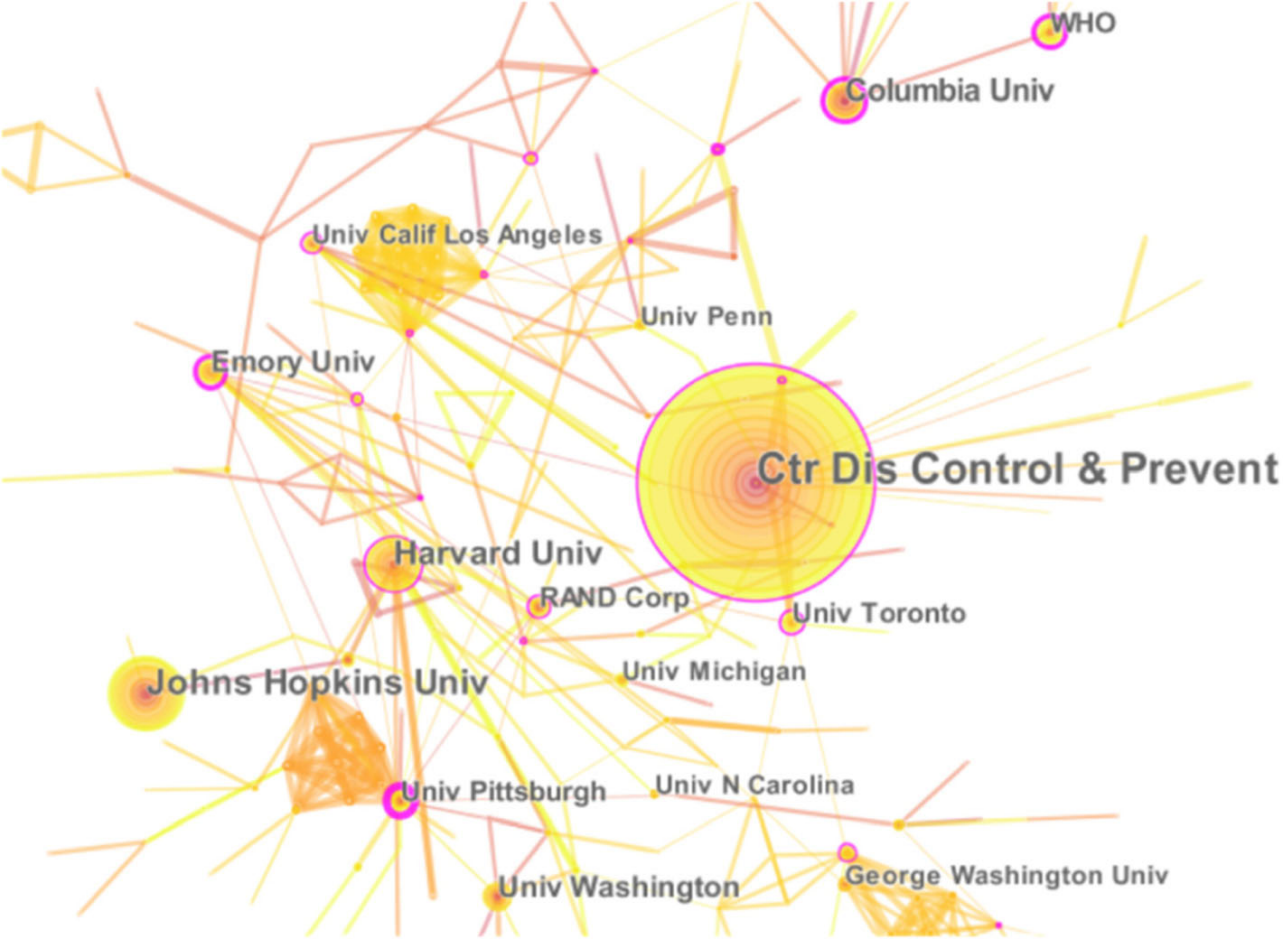
Co-institution network of international database. Different colors represent different time slices. The colors of rings of a circle are corresponding to the year. The diameter of each circle represents the number of the institution’s articles. The link strength between two nodes means the intensity of institution cooperation. The purple circles represent the high betweenness centralities

**Fig. 6:**
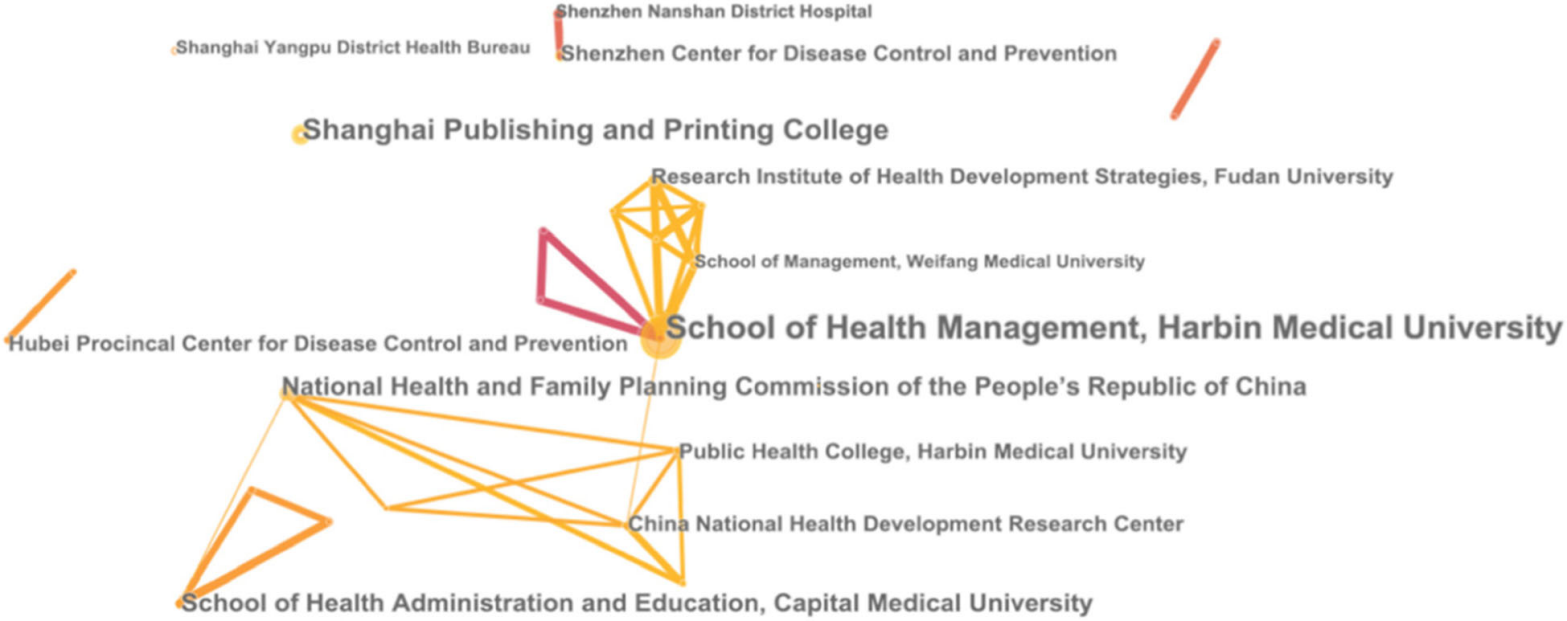
Co-institution network of Chinese database. Different colors represent different time slices. The colors of rings of a circle are corresponding to the year. The diameter of each circle represents the number of the institution’s articles. The link strength between two nodes means the intensity of institution cooperation

Similar to the co-author situation, many institutions have studied PHEM (Figs. 5 and 6). The top-ranked item by international citation count was the Centers for Disease Control and Prevention (Ctr Dis Control & Prevent.), with citation counts of 255, which means that the institution publishes the largest number of articles in this field. The second one was Johns Hopkins University (Johns Hopkins Univ.), with citation counts of 92, which means that Johns Hopkins University had the largest number of articles published among the universities in this field. Johns Hopkins was followed by Harvard University (Harvard Univ.), Columbia University (Columbia Univ.), and so on. The above analysis showed that the CDC and universities were the leading institutions to study international PHEM. The centrality ranked item was CDC (0.50). Then, Johns Hopkins University (0.21), Harvard University (0.16), University of Washington (Univ Washington., 0.13), University of Toronto (Univ Toronto., 0.10), University of Pittsburgh (Univ Pittsburgh, 0.10), and Boston University (Boston Univ., 0.10) followed. The centrality of all these nodes was no less than 0.1 with the purple circle, which meant that they were the institutions with higher publication quality and the leading institutions in this field. The top-ranked item by citation count in the Chinese database was the School of Health Management, Harbin Medical University, with a citation count of 20, followed by Shanghai Publishing and Printing College (9), National Health and Family Planning Commission of the People’s Republic of China (7), School of Health Administration and Education, Capital Medical University (6), etc. The top-ranked institution by centrality was the School of Health Management, Harbin Medical University, with a centrality of 0.04. The second one was the China National Health Development Research Center, with a centrality of 0.03. Thus, the School of Health Management, Harbin Medical University, is a leader in the field of research in China. In comparison, CDC had the largest publication volume and influence in the international field, while universities instead of health institutions dominated the field of research in China (Additional file 1: Table S3, Table S4, and Figure S2).

### Co-country

Table 1 summarizes the top 10 countries in the count and centrality of international PHEM research. We can see that the development of PHEM research differs among countries, and it is mainly centralized in the USA, Canada, England, Australia, People’s Republic of China, Switzerland, Italy, Sweden, Japan, and the Netherlands. According to the income levels from the World Bank [42], these were all high-income countries except China. Geographically, half of them are centralized in Europe. Among these countries, the USA was the most productive, far ahead of the rest in this field, and its centrality was the largest. This showed that in the field of PHEM, the USA carries out the most studies, and their studies were more advanced. Although China ranks fifth on this list, its centrality was only 0.03. Therefore, these results indicate that Chinese scholars could publish some internationally recognized articles in the field, which would offer an advantage in quantity; however, they need to improve their article quality. It is worth noting that although the number of articles from Switzerland (44) and Sweden (34) was much lower than that of the USA, the articles’ centrality of these two countries was more than 0.10, which showed that the quality of the articles was still high.

**Table 1:** Top 10 countries in the count and centrality of international database

Count	Country	Centrality	Country
1557	USA	0.67	USA
138	Canada	0.32	England
131	England	0.16	Switzerland
114	Australia	0.16	Sweden
95	P. R. China	0.11	Canada
44	Switzerland	0.08	Germany
34	Italy	0.06	Australia
34	Sweden	0.06	India
30	Japan	0.04	Netherlands
27	Netherlands	0.03	P. R. China

*Abbreviation*: *P. R. China* People’s Republic of China

### Co-word network analysis

#### Keywords co-occurrence network analysis

**Fig. 7:**
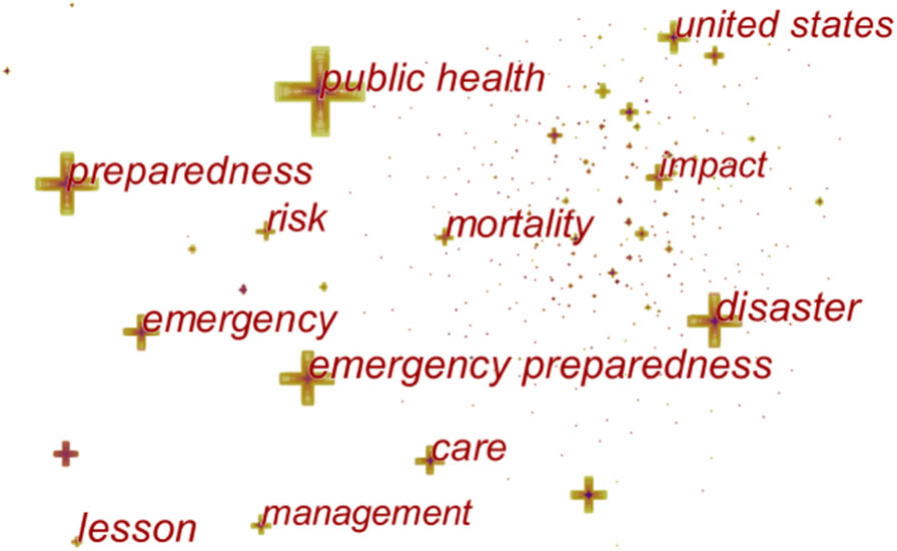
Keywords co-occurrence network of international database. The colors of crosses of a circle are corresponding to the year. The size of nodes represent research frequency of the keyword

**Fig. 8:**
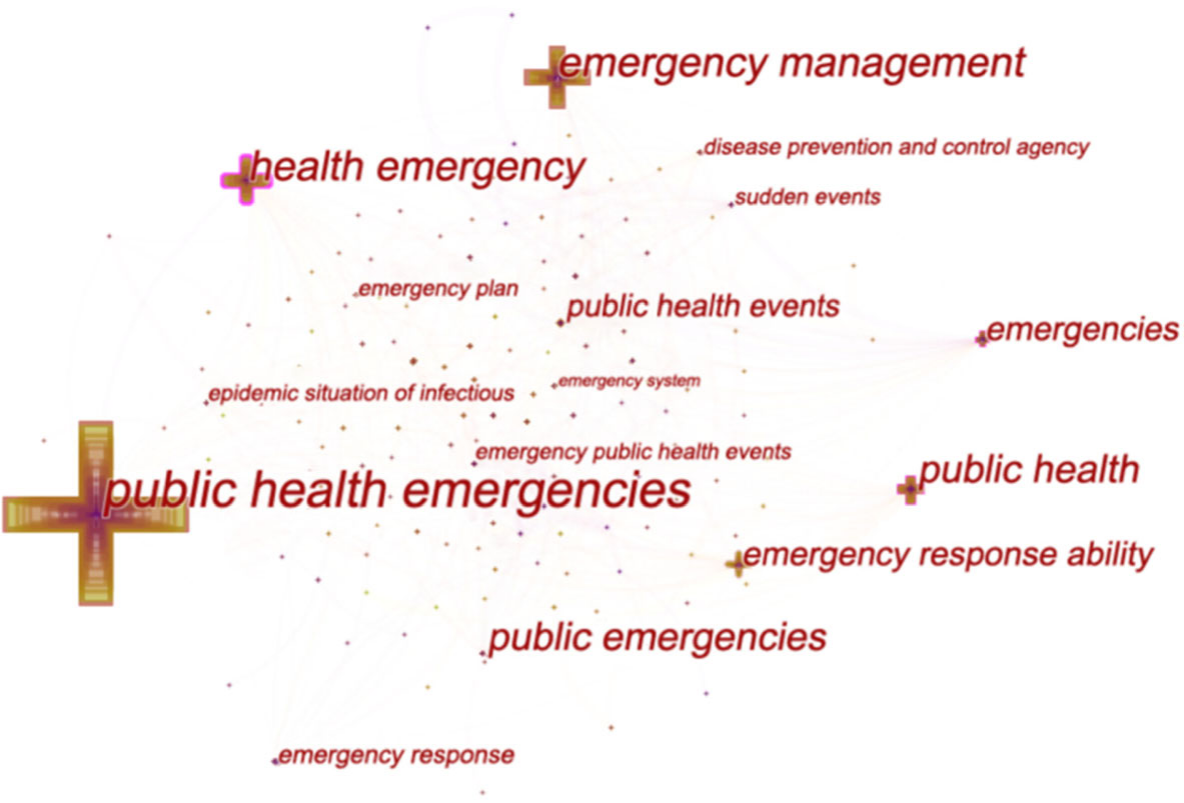
Keywords co-occurrence network of Chinese database. The colors of crosses of a circle are corresponding to the year. The size of nodes represent research frequency of the keyword

Generally, keywords represent the research hotspots, which represent topics of wide concern for researchers in this field. Figure 7 shows that the top 10 keywords ranked by citation count for the international database were public health (297), preparedness (215), emergency preparedness (191), disaster (187), health (142), emergency (133), care (128), United States (121), bioterrorism (102), and impact (96). The top ten centralities were emergency department (0.22), prevalence (0.18), surveillance (0.17), knowledge (0.15), public health preparedness (0.15), children (0.14), management (0.14), trauma (0.13), simulation (0.13), and policy (0.11). The centrality for all of them was more than 0.10, which meant they had more influence than other keywords. Figure 8 shows that the top 10 keywords of Chinese PHEM research were public health emergencies (394), emergency management (154), health emergency (116), public health (101), emergencies (84), emergency response ability (78), public health events (58), emergency mechanism (33), emergency disposal (28), and public emergencies (27). Nodes with a centrality over 0.1 were health emergency (0.45), public health (0.32), public health emergencies (0.30), emergency management (0.26), emergencies (0.23), public health events (0.13), and emergency response ability (0.12). Comparing the distribution of keywords in international and Chinese countries, we can see that international studies mainly focus on emergency preparedness and monitoring for public health events, while Chinese research mainly focuses on analysis and disposition (Additional file 1: Table S5, Table S6, and Figure S3).

#### Burst detection analysis

**Fig. 9:**
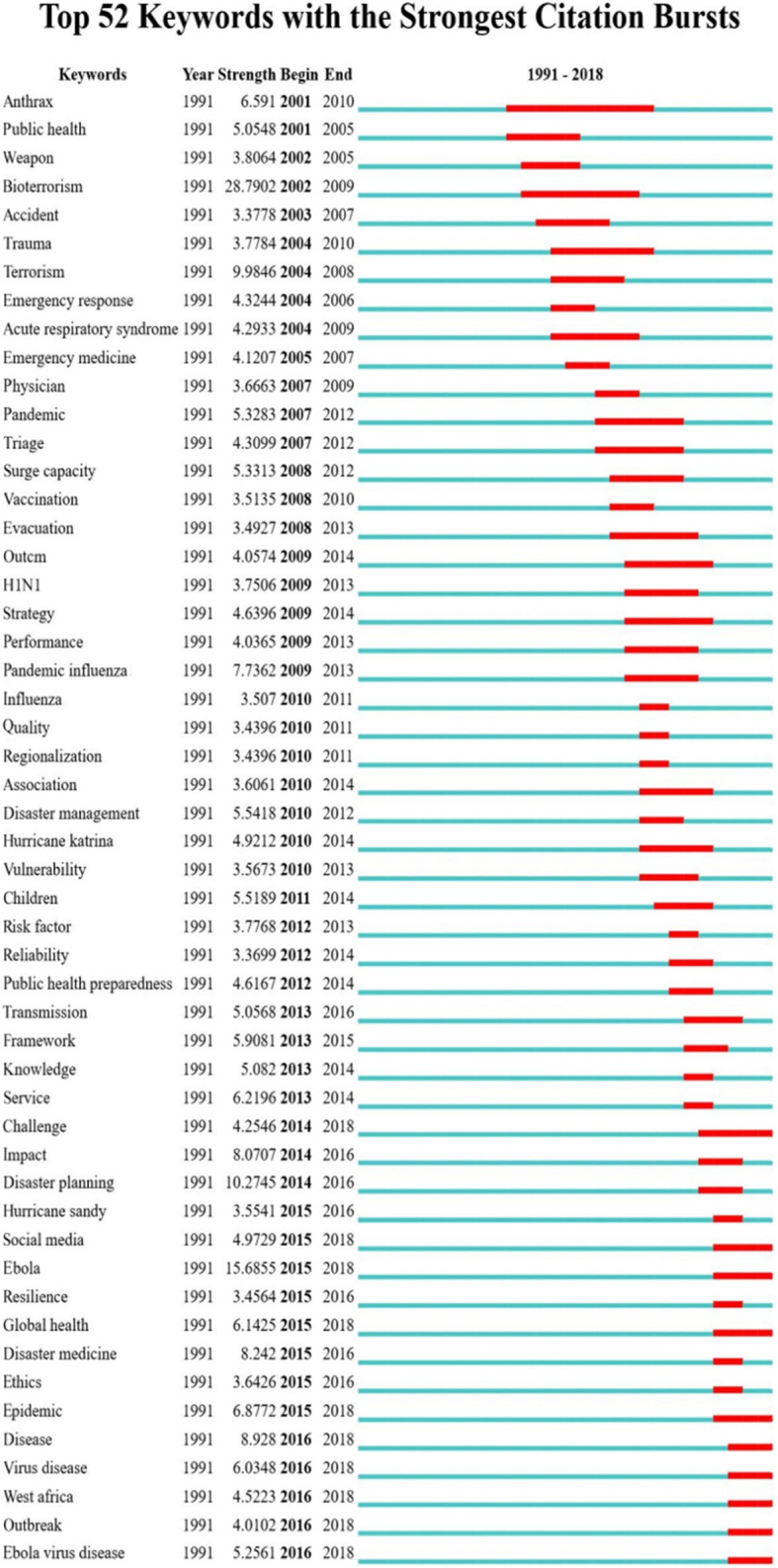
Keywords with the strongest citation bursts of international database. The strength represents the degree of the burst. The begin and end represent the boundaries of the time period of the burst. The blue line is the time interval, the red line segment is the duration of the burst for one keyword

**Fig. 10:**
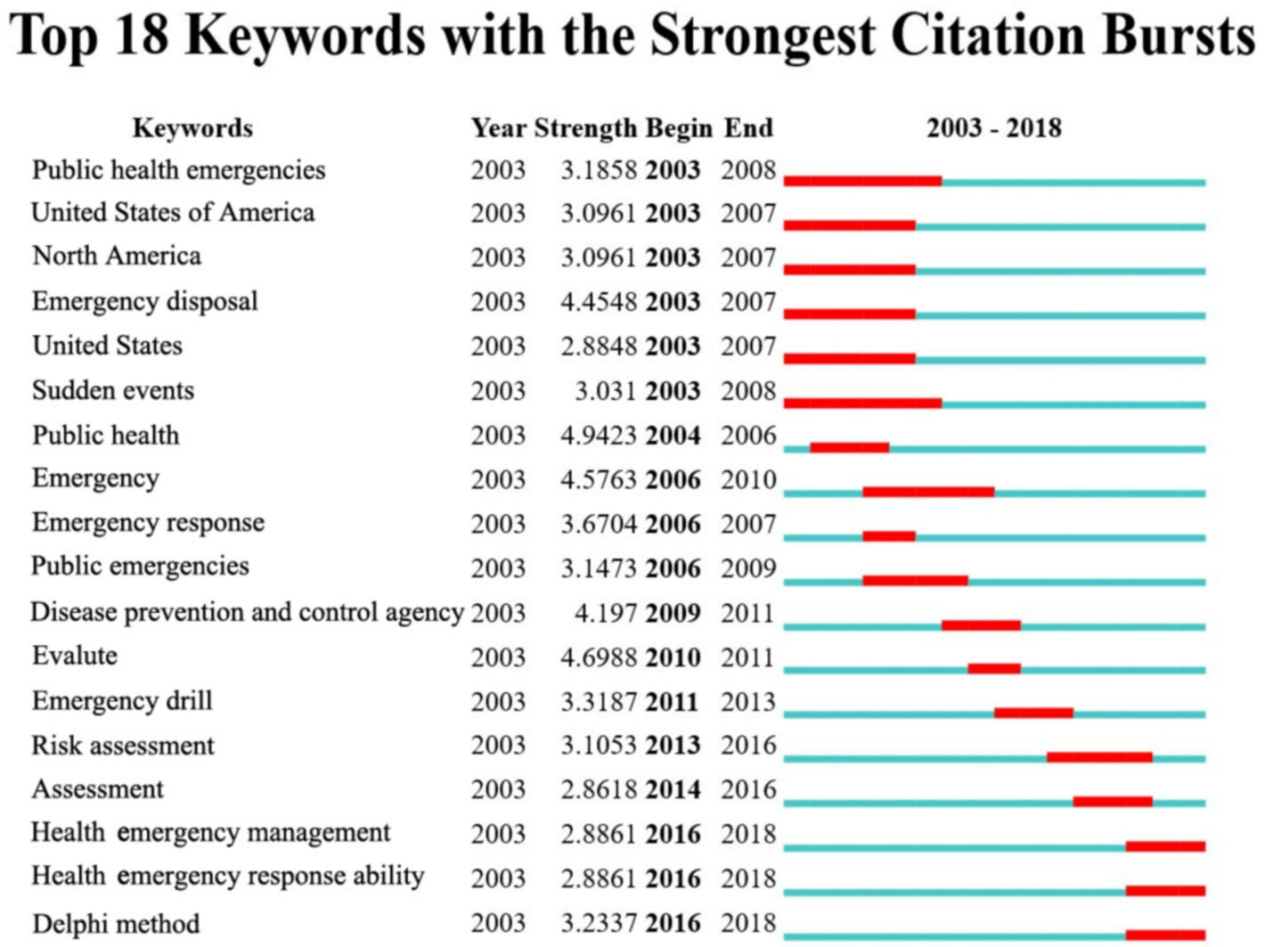
Keywords with the strongest citation bursts of Chinese database. The strength represents the degree of the burst. The begin and end represent the boundaries of the time period of the burst. The blue line is the time interval, the red line segment is the duration of the burst for one keyword

Figures 9 and 10 illustrate the keywords with the strongest citation burst for international and Chinese databases. The international keywords detected 52 burst words, while the Chinese data detected 18 burst words. The research frontier of international PHEM included anthrax, public health, weapon, bioterrorism, accident, trauma, terrorism, emergency response, and so on. Among these words, bioterrorism (28.7902) was the strongest burst keyword during the period between 2002 and 2009. Then, was Ebola (15.6855, 2015–2018), disaster planning (10.2745, 2014–2016), and terrorism (9.9846, 2004–2008). All these findings reflect a greater attention to this study topic and better exemplify the study front in this period. From the perspective of the time development sequence, international research on PHEM began with terrorism and bioterrorism. After that, disaster planning and emergency preparedness became a new research frontier (2004–2014). In recent years (2015–2018), epidemics and infectious diseases have become the focus of study. The research frontier of Chinese PHEM included public health emergencies, United States of America, North America, emergency disposal, united states, sudden events, public health, emergency, emergency response, public emergencies, disease prevention and control agency, evaluate, emergency drill, risk assessment, assessment, health emergency management, health emergency response ability, and the Delphi method. During 2003–2008, Chinese research on PHEM was in its infancy. The main research frontiers were public health emergencies and America. It was mainly at the stage of the formation of the Chinese theory of this field. By learning from the experience of the USA in dealing with health emergencies, scholars began the study of PHEM in China. Then, Chinese scholars began to study the links involved in the disposal of public health emergencies, such as response, organization, evaluation, exercise, and evaluation of PHEM. After 2016, health emergency management, health emergency response ability, and the Delphi method became the new research front. In contrast with Chinese research on PHEM, international research often relates to timely issues, while China focuses on the management procedures (Additional file 1: Figure S4).

To further explain the above research hotspots, the top 8 cited articles are shown in Tables 2 and 3. The top 8 ranked articles by citation for international databases appeared in 2005, 2007, 2009, 2014, 2015, and 2016. The article *Elevated blood lead levels in children associated with the flint drinking water crisis: a spatial analysis of risk and public health response* was the most cited (372) international art*icle* [43] followed by *The 2006 California Heat Wave: Impacts on Hospitalizations and Emergency department visit*s [44]. In terms of the time distribution, the research on international bioterrorism started first [45], followed by recommendations for health emergency response teams and health incident management [46, 47]. In recent years, the causes and disposal of public health events have been the hotspots of international attention [43, 48–50]. This was basically consistent with the analysis results of the above research keywords. The top 8 Chinese cited articles of PHEM appeared in 2003, 2004, 2009, and 2011. Table 3 shows us that the most frequently cited Chinese article was *Emergency logistics* [51], written by Zhongwen Ou, Huiyun Wang, and Dali Jiang et al. with a frequency of an astonishing 473. This was followed by Kaibin Zhong’s article *Review and prospect: construction of emergency management system in China* [52]. The next three articles, *Legislative situation and characteristics of China’s emergency law* [53], *Legal construction of public emergency response in China: legal construction task proposed by SARS crisis management practice* [54], and *The realistic subject of administrative rule of law in public emergency management* [55], mainly researched the legal construction of PHEM in China. They were all published in 2003, proving that China mainly carried out research on the legal construction of PHEM during that time. In other words, PHEM in China starts with legal system construction. In 2004, Chinese scholars began to establish a knowledge domain for health emergency response and disposition. By using extensive literature for reference, Chinese scholars began to research international PHEM in 2011.

**Table 2:** Top 8 cited articles of international database

Count	Author	Title	Journal	Year
372	Mona Hanna-Attisha, Jenny LaChance, Richard Casey Sadler, et al.	Elevated blood lead levels in children associated with the flint drinking water crisis: a spatial analysis of risk and public health response	American Journal of Public Health	2016
366	Kim Knowlton, Miriam Rotkin-Ellman, Galatea King, et al.	The 2006 California heat wave: impacts on hospitalizations and emergency department visits	Environmental Health Perspectives	2009
365	Karin Stettler, Martina Beltramello, Diego A. Espinosa, et al.	Specificity, cross-reactivity, and function of antibodies elicited by Zika virus infection	Science	2016
316	Nathalie Embriaco, Elie Azoulay, Karine Barrau, et al.	High level of burnout in intensivists—prevalence and associated factors	American Journal of Respiratory and Critical Care Medicine	2007
305	Salim S. Abdool Karim, Gavin J. Churchyard, Quarraisha Abdool Karim, et al.	HIV infection and tuberculosis in South Africa: an urgent need to escalate the public health response	Lancet	2009
286	Daniel P. Aldrich, Michelle A. Meyer	Social Capital and Community Resilience	American Behavioral Scientist	2014
238	Lawrence M. Wein, Yifan Liu	Analyzing a bioterror attack on the food supply: the case of botulinum toxin in milk	Proceedings of the National Academy of Sciences of the United States of America	2005
226	Philip J. Peters, Pamela Pontones, Karen W. Hoover, et al.	HIV infection linked to injection use of oxymorphone in Indiana, 2014-2015	The New England Journal Of Medicine	2016

**Table 3:** Top 8 cited articles of Chinese database

Count	Author	Title	Journal	Year
473	Zhongwen Ou, Huiyun Wang, Dali Jiang, et al.	Emergency logistics	Journal of Chongqing University (Natural Science Edition)	2004
177	Kaibin Zhong	Review and prospect: construction of emergency management system in China	CASS Journal of Political Science	2009
122	Jihong Mo	Legislative situation and characteristics of China’s emergency law	Legal Forum	2003
102	Yuchuan Mo	Legal construction of public emergency response in China: legal construction task proposed by SARS crisis management practice	Journal of Renmin University of China	2003
94	Yuchuan Mo	The realistic subject of administrative rule of law in public emergency management	Jurists Review	2003
77	Yifeng Yang, Chenfang Fan, Guangwen Cao	Emergency management in immediate response to emergent public health event in China	Academic Journal of Second Military Medical University	2004
69	Lexuan Luo, Zhanchun Feng, Jian Zhang	Research on the hospital function of response to public health emergency	Chinese Hospital Management	2004
65	Liping Fan, Qinghua Zhao	The status quo of emergency management system for sudden public health events in America and Japan and its enlightenment	Chinese Nursing Research	2011

## Discussion

International research on PHEM occurred earlier than the Chinese research, and it has been growing over time. This means that international scholars have paid increasing attention to PHEM. In 1991, the first article on PHEM was written by Richard L. Siegel and was titled *Code 9: a systematic approach for responding to medical emergencies occurring in and around a hospital* [56]. It mentioned the need for an organized system to respond to such emergencies involving patients, visitors, local community residents, and hospital employees, both inside the hospital and on the grounds surrounding the building. He recommended the establishment of a systematic emergency response system in all health care institutions. Since then, academia has begun to pay attention to emergency management of public health incidents. The number of international articles is increasing gradually, reaching the maximum in 2017, and it is expected to continue to grow in the next 3 years. The development of PHEM in China shows a fluctuating pattern. The occurrence of public health emergencies in the 10 years from 2006 to 2016 showed a general trend of first rising and then slowly declining. It is likely related to the number of significant events that occur in each year [57]. The severe acute respiratory syndrome (SARS) epidemic in 2003 resulted in significant increases in both the amount of research and articles on emergency management [7]. The number of articles reached a small climax in 2008. Events such as the Wenchuan earthquake and the southern snow disaster occurred in that year. The maximum was in 2013, with human infection from H7N9, the Ya’an earthquake, and death from a hepatitis B vaccine occurring that year. Moreover, 10 years after the SARS outbreak, some authors compared the development of PHEM in China over the 10-year period. The first Chinese article on PHEM was written by Tiewu Jia and was titled *Capacity-building for public health emergency response to disasters* (2003) [58]. This article was published during the epidemic of SARS. In 2003, China did not establish a network and echelon PHEM system. The author combined the development of emergency management, reform of health and epidemic prevention institutions, and discussed the capacity building of public health emergency response. It is helpful for the social function orientation of the disease control center and the improvement of disease prevention ability. Although the number of Chinese articles decreased in the following years, it remained above 48. In summary, the above analysis shows that PHEM is still a timely topic.

From the perspective of cooperative networks, we find that there is more cooperation among Chinese authors but less cooperation among authors from different institutions. The cooperation between different research institutions is believed to be highly effective in facilitating high-level and fruitful research, which can also help develop the research field into a more established area [59]. Therefore, Chinese scholars should strengthen cooperation between different institutions. The research institution focus on PHEM mainly comes from universities and health institutions, while Chinese institutions have regional differences. Reasons include the following: the western region had poor fiscal capacity, a limited personnel size, and an inadequate stockpile in terms of working budget, timely reserves, and prompt delivery [60]. As a leader in international PHEM, the CDC has begun to help other entities strengthen their capacity, recognition, and technical expertise to strengthen their PHEM capacity [61]. Additionally, some other health institutions, such as the WHO, have promoted development in this field. In 2005, the 58th World Health Assembly (WHA) adopted the revised International Health Regulations, which instructed the WHO member states to collaboratively confront public health emergencies of global significance [5, 17, 62]. Universities have undertaken the scientific task of PHEM, and they have conducted in-depth research on it in China. The Chinese CDC has carried out more disease prevention and control services, but its scientific research ability is weak. The country network analysis shows obvious differences in regional and economic development levels for PHEM. Those countries with more developed health emergency management systems are the most high-income ones. Geographically, most of these countries are concentrated in Europe, where the numbers of publications and citations are also significantly higher [60]. The USA, the UK, Japan, and other countries have constantly built and improved their PHEM systems, which have become a comprehensive management network.

Co-word analysis of PHEM international research is more complex, extensive, and multidimensional. It reflects some of the major ideas of this research. Based on these ideas, scholars mainly focused on emergency preparedness and monitoring of public health events. From the perspective of Chinese PHEM development, it has gone through a process from theory to preparation, disposition, response, evaluation, organization, and discussion. That is, the main contents of China’s health emergency management include the prevention and preparation of health emergencies as well as the key links of disposition, evaluation, and management, system construction, personnel training, and so on. The development of the whole discipline is therefore systematic and clear. The keywords with the strongest citation burst for international research on PHEM started with terrorism and bioterrorism [63], followed by disaster planning and emergency preparedness. In recent years, epidemics and infectious diseases have become the new research frontier. From the perspective of the whole development context, international research on PHEM has been related to current affairs hotspots, such as terrorism, which may have originated from the 911 incident, and epidemics, which may be related to the epidemic of infectious diseases caused by viruses and bacteria such as the Ebola virus. The study of PHEM in China is a process from theory formation to practice discussion, involving many links of management. During 2003–2008, Chinese scholars focused on health emergency response and disposition. After that, Chinese scholars began to learn more about foreign PHEM models. Some new methods have gradually been applied to Chinese PHEM in recent years.

The top-ranked articles by citation for the international knowledge domain of PHEM appeared in 2005, 2007, 2009, 2014, 2015, and 2016. In 2005, Lawrence M. Wein [45] developed a mathematical model of a cow-to-consumer supply chain to reduce bioterrorism events. Once again, it shows that international emergency management research is based on terrorism and bioterrorism. In 2007, Nathalie Embriaco focused on the working condition of emergency management personnel [46]. Kim Knowlton [44] and Salim S. Abdool Karim [47] mentioned the emergency department. The above three articles are all about the factors involved in health emergency management. The remaining articles analyze the specific events involving the mechanism, response, and recovery [43, 48–50]. From the above analysis, it can be seen that terrorism, emergency response and health incident management, and the disposition of public health events are the hotspots of international attention. *Legislative situation and characteristics of China’s emergency law* [53], *Legal construction of public emergency response in China: legal construction task proposed by SARS crisis management practice* [54], and *The realistic subject of administrative rule of law in public emergency management* [55] were published in 2003. All three articles discussed the problems existing in the construction of the administrative legal system under the background of SARS. After that, three articles were published in 2004, mainly studying the mechanism and structure of PHEM in China. This research proposed the need to establish the emergency response mechanism for PHEM and establish emergency structure construction as soon as possible. In 2009, Kaibin Zhong wrote the article *Review and prospect: construction of emergency management system in China* [52]. He elaborated on the core contents of Chinese PHEM construction, including emergency plans, emergency structures, emergency mechanisms, and legal systems. China’s PHEM integrates emergency systems, emergency mechanisms, and legal systems in an all-round way, which is characterized by comprehensiveness, institutionalization, openness, and guarantees. In 2011, *The status quo of emergency management system for sudden public health events in America and Japan and its enlightenment* [64] was published, showing that China has been learning the theory and experience of PHEM from some advanced countries. From the above analysis, it can be seen that the legal system, mechanism, and structure, system, and learning from abroad are the theoretical guidance for Chinese PHEM in the past 30 years.

Admittedly, there are some limitations to this study. First, the conclusions drawn from this study were based on only two large literature retrieval libraries. Other databases, such as Embase and Springer Link, were not studied. Not being able to search all the literature in this field may lead to incomplete retrieval results. Second, CiteSpace has some shortcomings in processing the results of the Chinese database; it cannot translate the result from Chinese into English directly. Third, there is a 1-year or longer time lag between our paper submission and its publication. The database articles may change during this time. Fourth, we conducted a comparison between Chinese and international databases similar to that performed in many other studies. It should be acknowledged that the two databases had different acceptance ratios, and this difference in data sources might lead to bias in the study results. In addition, we categorized English articles focusing on China as being part of the international database and did not analyze them alone. Although only a small part of the total, this may have created some deficiencies. This limitation may constitute an object of future studies, namely, those analyzing the differences between English papers focusing on China vs. Chinese papers.

## Conclusions

In summary, we selected two large retrieval library documents to define the PHEM domain and detected the research status and the trends related to it from 1991 to 2018. According to the analyses, the conclusions are as follows. In the next 3 years, the number of international PHEM articles will continue to increase, while the number of Chinese articles will decline. Chinese scholars show less cooperation among different organizations. There are differences in regional and economic distribution between international and Chinese cooperation networks. China focuses on the east regionally, while developed countries and European countries have a more international focus. International research often relates to timely issues, mainly by focusing on emergency preparedness and monitoring of public health events, while China focuses on public health emergencies and their resolution. The international research on PHEM begins with terrorism and bioterrorism, followed by disaster planning and emergency preparedness, and emerging infectious diseases. China uses SARS as the research background and the legal system construction as the research starting point, which is followed by the mechanism and structure, system, and training abroad.

## Supplementary information

**Additional file 1: Table S1.** Top 10 authors in the published volume and centrality of international database. **Table S2.** Top 10 authors in the published volume and centrality of Chinese database. **Table S3.** Top 10 institutions in the published volume and centrality of international database. **Table S4.** Top 10 institutions in the published volume and centrality of Chinese database. **Table S5.** Top 10 keywords ranked by citation counts and centrality of international database. **Table S6.** Top 10 keywords ranked by citation counts and centrality of Chinese database. **Figure S1.** Co-author network of Chinese database. **Figure S2.** Co-institution network of Chinese database. **Figure S3.** Keyword co-occurrence network of Chinese database. **Figure S4.** Keywords with the strongest citation bursts of Chinese database.

## Data Availability

All data generated or analyzed during this study are included in this published article and its supplementary information files.

## Abbreviations

EM-DAT: Emergency events database
COVID-19: Coronavirus disease 2019
PHEM: Public health emergency management
CDC: Center for Disease Prevention and Control
WoS: Web of Science
SCI-E: Science Citation Index Expanded
SSCI: Social Sciences Citation Index
A&HCI: Arts & Humanities Citation Index
CNKI: China National Knowledge Infrastructure
WHO: World Health Organization
HIV: Human immunodeficiency virus
AIDS: Acquired immune deficiency syndrome
SARS: Severe acute respiratory syndrome
WHA: World Health Assembly
