# Supplementary material for: A comparative study of international and Chinese public health emergency management from the perspective of knowledge domains mapping
Source: Environ Health Prev Med. 2020 Oct 2;25:57. doi: 10.1186/s12199-020-00896-z (PMC7531067; doi:10.1186/s12199-020-00896-z)

**A Comparative Study of International and Chinese Public Health Emergency Management from the Perspective of Knowledge Domains Mapping**

Supplemental data

**Table S1** Top 10 authors in the published volume and centrality of international database

| **Count** | **Author** | **Centrality** | **Author** |
| --- | --- | --- | --- |
| 20 | Daniel J. Barnett  (DANIEL J BARNETT) | 0.06 | Frederick M. Burkle  (FREDERICK M) |
| 17 | Elena Savoia  (ELENA SAVOIA) | 0.06 | Task Force for Pediat Emergency Mass Critical Care  (TASK FORCE PEDIAT EMERGENCY MASS C CA) |
| 17 | Lainie Rutkow  (LAINIE RUTKOW) | 0.06 | James G. Hodge  (JAMES G) |
| 14 | Jonathan M. Links  (JONATHAN M LINKS) | 0.05 | Daniel J. Barnett  (DANIEL J BARNETT) |
| 14 | Frederick M. Burkle  (FREDERICK M) | 0.03 | Kasisomayajula Viswanath  (KASISOMAYAJULA VISWANATH) |
| 12 | Michael A. Stoto  (MICHAEL A STOTO) | 0.03 | Robert J. Blendon  (ROBERT J BLENDON) |
| 12 | Niranjan Kissoon  (NIRANJAN KISSOON) | 0.03 | Emily B. Kahn  (EMILY B KAHN) |
| 10 | Edbert B. Hsu  (EDBERT B HSU) | 0.02 | Elena Savoia  (ELENA SAVOIA) |
| 9 | Nicole Lurie  (NICOLE LURIE) | 0.02 | Jonathan M. Links  (JONATHAN M LINKS) |
| 9 | Carol B. Thompson  (CAROL B THOMPSON) | 0.02 | Edbert B. Hsu  (EDBERT B HSU) |

**Table S2** Top 10 authors in the published volume and centrality of Chinese database

| **Count** | **Author** | **Centrality** | **Author** |
| --- | --- | --- | --- |
| 22 | 吴群红 (Qunhong Wu) | 0.02 | 郝艳华 (Yanhua Hao) |
| 17 | 郝艳华 (Yanhua Hao) | 0.02 | 宁宁 (Ning Ning) |
| 11 | 韩锋 (Feng Han) | 0.01 | 吴群红 (Qunhong Wu) |
| 10 | 宁宁 (Ning Ning) | 0.01 | 刘颖 (Ying Liu) |
| 10 | 王亚东 (Yadong Wang) | 0.00 | 韩锋 (Feng Han) |
| 8 | 康正 (Zheng Kang) | 0.00 | 王亚东 (Yadong Wang) |
| 6 | 刘颖 (Ying Liu) | 0.00 | 康正 (Zheng Kang) |
| 6 | 马金成 (Jincheng Ma) | 0.00 | 马金成 (Jincheng Ma) |
| 5 | 焦明丽 (Mingli Jiao) | 0.00 | 焦明丽 (Mingli Jiao) |
| 5 | 梁立波 (Libo Liang) | 0.00 | 梁立波 (Libo Liang) |

**Table S3** Top 10 institutions in the published volume and centrality of international database

| **Count** | **Institution** | **Centrality** | **Institution** |
| --- | --- | --- | --- |
| 255 | Centers for Disease Control and Prevention  (Ctr Dis Control & Prevent) | 0.50 | Centers for Disease Control and Prevention  (Ctr Dis Control & Prevent) |
| 92 | Johns Hopkins University  (Johns Hopkins Univ) | 0.21 | Johns Hopkins University  (Johns Hopkins Univ) |
| 71 | Harvard University  (Harvard Univ) | 0.16 | Harvard University  (Harvard Univ) |
| 51 | Columbia University  (Columbia Univ) | 0.13 | University of Washington  (Univ Washington) |
| 49 | Emory University  (Emory Univ) | 0.10 | University of Toronto  (Univ Toronto) |
| 42 | University of Washington  (Univ Washington) | 0.10 | University of Pittsburgh  (Univ Pittsburgh) |
| 35 | World Health Organization  (WHO) | 0.10 | Boston University  (Boston Univ) |
| 31 | RAND Corporation  (RAND Corp) | 0.09 | Columbia University  (Columbia Univ) |
| 30 | University of Toronto  (Univ Toronto) | 0.09 | World Health Organization  (WHO) |
| 30 | University of Pittsburgh  (Univ Pittsburgh) | 0.08 | Emory University  (Emory Univ) |

**Table S4** Top 10 institutions in the published volume and centrality of Chinese database

| **Count** | **Institution** | **Centrality** | **Institution** |
| --- | --- | --- | --- |
| 20 | 哈尔滨医科大学卫生管理学院(School of Health Management, Harbin Medical University) | 0.04 | 哈尔滨医科大学卫生管理学院(School of Health Management, Harbin Medical University) |
| 9 | 上海出版印刷高等专科学校(Shanghai Publishing and Printing College) | 0.03 | 卫生部卫生发展研究中心  (China National Health Development Research Center) |
| 7 | 国家卫生和计划生育委员会(National Health and Family Planning Commission of the People’s Republic of China) | 0.02 | 国家卫生和计划生育委员会(National Health and Family Planning Commission of the People’s Republic of China) |
| 6 | 首都医科大学卫生管理与教育学院  (School of Health Administration and Education, Capital Medical University) | 0.02 | 首都医科大学卫生管理与教育学院  (School of Health Administration and Education, Capital Medical University) |
| 6 | 深圳市疾病预防控制中心(Shenzhen Center for Disease Control and Prevention) | 0.00 | 上海出版印刷高等专科学校(Shanghai Publishing and Printing College) |
| 5 | 卫生部卫生发展研究中心  (China National Health Development Research Center) | 0.00 | 深圳市疾病预防控制中心(Shenzhen Center for Disease Control and Prevention) |
| 5 | 湖北省疾病预防控制中心  (Hubei Procincal Center for Disease Control and Prevention) | 0.00 | 湖北省疾病预防控制中心  (Hubei Procincal Center for Disease Control and Prevention) |
| 5 | 哈尔滨医科大学公共卫生学院(Public Health College,  Harbin Medical University) | 0.00 | 哈尔滨医科大学公共卫生学院(Public Health College,  Harbin Medical University) |
| 4 | 潍坊医学院管理学院  (School of Management, Weifang Medical University) | 0.00 | 潍坊医学院管理学院  (School of Management, Weifang Medical University) |
| 4 | 复旦大学卫生发展战略研究中心(Research Institute of Health Development Strategies, Fudan University) | 0.00 | 复旦大学卫生发展战略研究中心(Research Institute of Health Development Strategies, Fudan University) |

**Table S5** Top 10 keywords ranked by citation counts and centrality of international database

| **Count** | **Keyword** | **Centrality** | **Keyword** |
| --- | --- | --- | --- |
| 297 | public health | 0.22 | emergency department |
| 215 | preparedness | 0.18 | prevalence |
| 191 | emergency preparedness | 0.17 | surveillance |
| 187 | disaster | 0.15 | knowledge |
| 142 | health | 0.15 | public health preparedness |
| 133 | emergency | 0.14 | children |
| 128 | care | 0.14 | management |
| 121 | United States | 0.13 | trauma |
| 102 | bioterrorism | 0.13 | simulation |
| 96 | impact | 0.11 | policy |

**Table S6** Top 10 keywords ranked by citation counts and centrality of Chinese database

| **Count** | **Keyword** | **Centrality** | **Keyword** |
| --- | --- | --- | --- |
| 394 | 突发公共卫生事件  (public health emergencies) | 0.45 | 卫生应急  (health emergency) |
| 154 | 应急管理  (emergency management) | 0.32 | 公共卫生  (public health) |
| 116 | 卫生应急  (health emergency) | 0.30 | 突发公共卫生事件  (public health emergencies) |
| 101 | 公共卫生  (public health) | 0.26 | 应急管理  (emergency management) |
| 84 | 突发事件  (emergencies) | 0.23 | 突发事件  (emergencies) |
| 78 | 应急能力  (emergency response ability) | 0.13 | 公共卫生事件  (public health events) |
| 58 | 公共卫生事件  (public health events) | 0.12 | 应急能力  (emergency response ability） |
| 33 | 应急机制  (emergency mechanism) | 0.09 | 突发公共事件  (public emergencies) |
| 28 | 应急处置  (emergency disposal) | 0.08 | 疾控机构  (disease prevention and control agency) |
| 27 | 突发公共事件  (public emergencies) | 0.06 | 应急预案  (emergency plan) |

**Figure S1.** Co-author network of Chinese database. Different colors represent different time slices. The size of node represents the number of articles by the author. The link strength between two nodes means the collaboration intensity between authors.


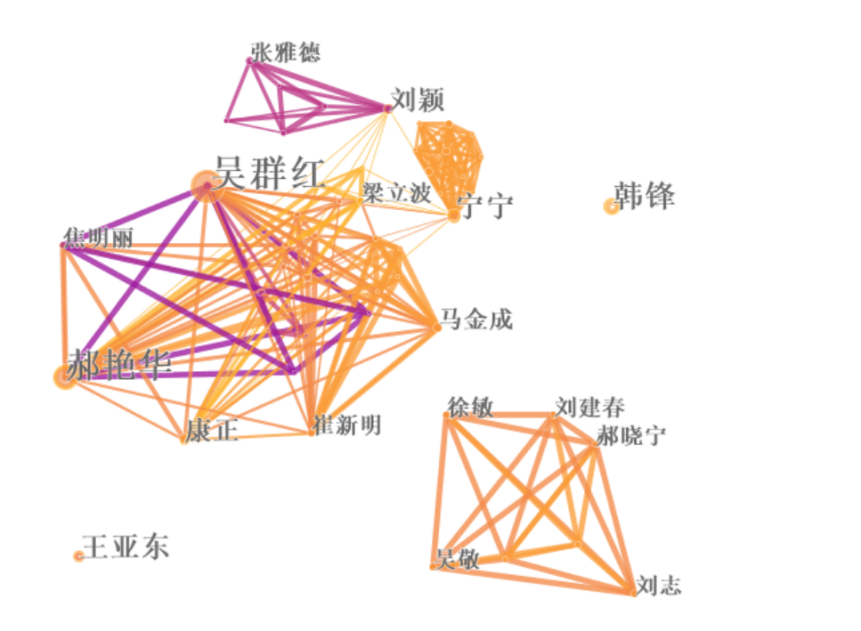


**Figure S2.** Co-institution network of Chinese database. Different colors represent different time slices. The colors of rings of a circle are corresponding to the year. The diameter of each circle represents the number of the institution’s articles. The link strength between two nodes means the intensity of institution cooperation.


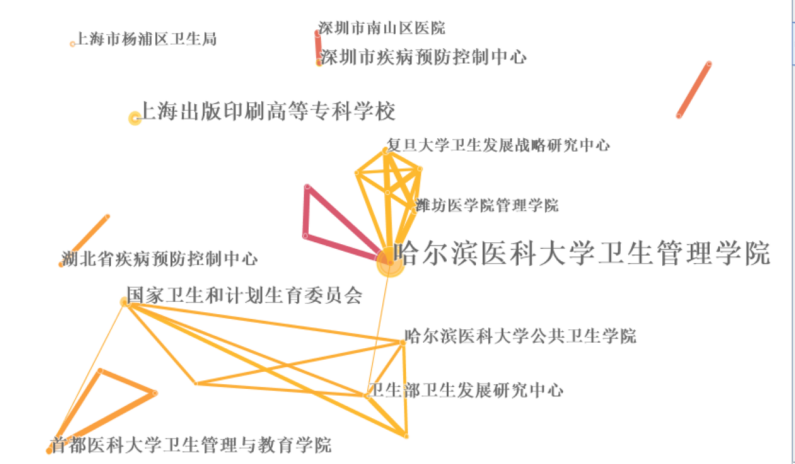


**Figure S3.** Keyword co-occurrence network of Chinese database. The colors of crosses of a circle are corresponding to the year. The size of nodes represent research frequency of the keyword.


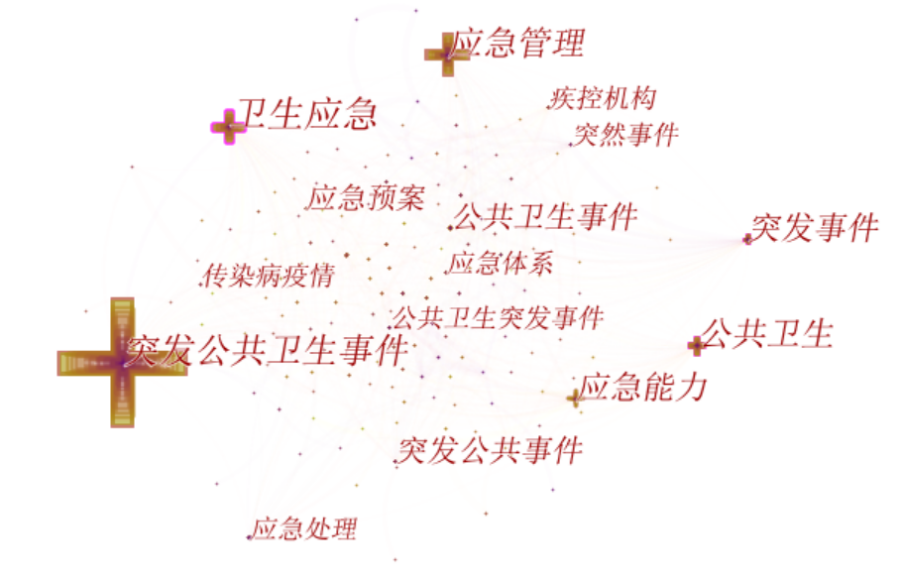


**Figure S4.** Keywords with the strongest citation bursts of Chinese database. The strength represents the degree of the burst. The begin and end represent the boundaries of the time period of the burst. The blue line is the time interval, the red line segment is the duration of the burst for one keyword.


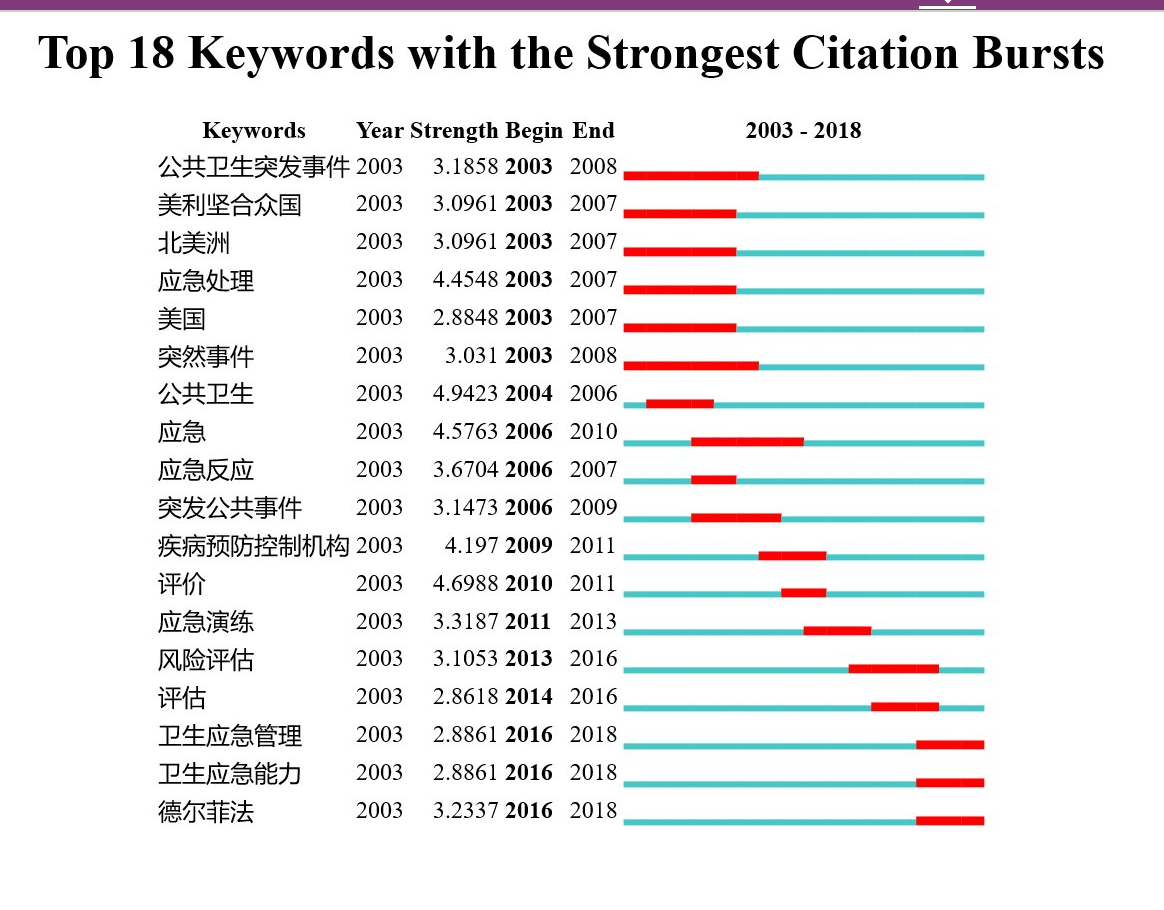

Supplement: Supplementary file 1 — Additional file 1: Table S1. Top 10 authors in the published volume and centrality of international database. Table S2. Top 10 authors in the published volume and centrality of Chinese database. Table S3. Top 10 institutions in the published volume and centrality of international database. Table S4. Top 10 institutions in the published volume and centrality of Chinese database. Table S5. Top 10 keywords ranked by citation counts and centrality of international database. Table S6. Top 10 keywords ranked by citation counts and centrality of Chinese database. Figure S1. Co-author network of Chinese database. Figure S2. Co-institution network of Chinese database. Figure S3. Keyword co-occurrence network of Chinese database. Figure S4. Keywords with the strongest citation bursts of Chinese database. [file 12199_2020_896_MOESM1_ESM.doc]
